# Neoagaro-Oligosaccharides Ameliorate Chronic Restraint Stress-Induced Depression by Increasing 5-HT and BDNF in the Brain and Remodeling the Gut Microbiota of Mice

**DOI:** 10.3390/md20110725

**Published:** 2022-11-18

**Authors:** Yan Zhuang, Runying Zeng, Xiao Liu, Longhe Yang, Zhuhua Chan

**Affiliations:** 1 Marine Biological Resources Development and Utilization Engineering Technology Innovation Center, Third Institute of Oceanography, Ministry of Natural Resources, Xiamen 361005, China; 2 Fujian Provincial Key Laboratory of Island Conservation and Development, Island Research Center, Ministry of Natural Resources, Pingtan 350400, China; 3 School of Advanced Manufacturing, Fuzhou University, Fuzhou 362200, China

**Keywords:** depression, NAOs, gut microbiota, 5-HT, BDNF, SCFAs, fecal microbiota transplantation

## Abstract

Neoagaro-oligosaccharides (NAOs) belong to the algae oligosaccharides. NAOs have been found to have diverse biological activities. However, the effects of NAOs on depression and their underlying mechanism have not been thoroughly studied. A chronic restraint stress (CRS)-induced C57BL/6J mouse model was used to assess the antidepressant effects of NAOs. Anxiety and depression behaviors were assessed by open field tests (OFT) and forced swimming tests (FST), while interleukin 18 (IL-18), 5-hydroxytryptamine (5-HT) and brain-derived neurotrophic factor (BDNF) were the molecular biomarkers of depression. Fecal microbiota transplantation (FMT) was performed. The results showed that NAO treatment significantly improved the body weight of depressed mice and reduced the central area time in the OFT and immobility time in the FST. NAO treatment decreased the levels of IL-18 in the serum and increased the levels of 5-HT in the serum and whole brain and of BDNF in the whole brain. NAO treatment mitigated the gut microbiota dysbiosis in the depressed mice and reversed the decreased levels of short-chain fatty acids (SCFAs) in the cecum of the depressed mice. FMT indicated that the gut microbiota is, indeed, linked to depression, which was reflected in the changes in weight gain and behaviors. In a word, NAOs effectively reversed the CRS-induced mice model of depression, which depended on the changes in the gut microbiota and SCFAs, as well as its modulation of 5-HT and BDNF.

## 1. Introduction

Depression is a common mental illness characterized by low mood, diminished interest, and a reduced sense of joy, with an incidence rate of 6.4% to 10.4% [1,2]. Depression reduces the quality of life of patients and decreases their life expectancy by ~10 years [3]. The global burden of disease caused by depression has been increasing in recent decades, and depression has become one of the leading causes of disability worldwide [4], affecting more than 264 million people [5]. In particular, the prevalence of depression and anxiety in adolescents has increased significantly, especially with the outbreak and continuation of the coronavirus disease 2019 (COVID-19) pandemic [6]. Neuropsychiatric symptoms (e.g., fatigue, depression) have been reported in individuals affected by COVID-19, suggesting that COVID-19 affects the central nervous system (CNS) [7,8]. One of the possible explanations for depression is the insufficient production of neurotrophic factors (e.g., brain-derived neurotrophic factor (BDNF)) in depressed individuals [9]. Meanwhile, experiments have shown that the serum interleukin-18 (IL-18) is a potential marker for the development of depression [10]. Drugs used for the treatment of depression include selective serotonin reuptake inhibitors (SSRIs), tricyclic antidepressants, and monoamine oxidase inhibitors. Even if SSRIs show great tolerability and high efficacy in their use in relation to other antidepressant classes [11], their use, particularly that of paroxetine, is associated with several side effects, including headaches, dizziness, weakness, difficulty concentrating, nervousness, forgetfulness, confusion, or sleepiness, leading, in some cases, to the termination of patients’ treatment. Despite the effectiveness of antidepressants, currently, much uncertainty still exists about the complete molecular mechanism of depression, so that it is highly challenging to identify new antidepressants [12].

Previous studies have discovered that an abnormal microbiota composition in the host gut may contribute to the pathogenesis of depression [13,14]. On the one hand, the consequences of gut microbiota dysbiosis are often associated with the dysregulation of the microbiota–host co-metabolism [15]. On the other hand, the construction of normal gut flora can effectively alleviate depression symptoms [16]. In one study, the transplantation of fecal microbiota from patients with depression to the intestines of germ-free (GF) mice could induce depression-like behavior [16]. Moreover, probiotics could counteract gut dysbiosis and produce antidepressant-like effects [17,18]. The current documentation supports the microbiota–gut–brain (MGB) axis, which refers to the network of connections involving multiple biological systems that enables bidirectional communication between the gut bacteria and brain. In addition, the MGB axis is important in maintaining the homeostasis of the gastrointestinal, CNS, and microbial systems of animals and plays a role in depression [19,20].

Neoagaro-oligosaccharides (NAOs), degraded from algae polysaccharides by agarases, are important natural substances with many bioactivities [21]. A few studies of NAOs reported their diverse biological activities, such as their antioxidative [22,23], anti-inflammatory [24,25,26,27,28,29], anti-obesity, and anti-diabetic [30] effects. Although NAOs cannot be digested by the host gastrointestinal tract, they can selectively stimulate the growth of two beneficial bacteria in the host’s intestinal tract, the bifidobacteria and lactobacilli, which improve host health. Several studies indicated the prebiotic effect of NAOs through in vivo experiments. In these studies, the structure of the intestinal flora in mice was improved after the ingestion of NAOs, and the proliferation of bifidobacteria and lactobacilli was promoted by the NAOs selectively, and the growth of harmful bacteria such as enterococci was inhibited [31]. The results further showed that NAOs can improve gut health through microbial remodeling. Notably, microbial remodeling has been closely associated with a dysfunctional “gut–brain axis” that underlies maladaptation to stress, neurogenesis impairment, and behavioral abnormality [32,33,34].

Here, we report that NAO treatment can ameliorate chronic restraint stress (CRS)-induced depression. In this study, 28 days of CRS induction led to slow weight gain in mice, and they exhibited anxiety- and depressive-like behavior. This was accompanied by an increase in the levels of IL-18 and a decrease in the levels of 5-hydroxytryptamine (5-HT) in the serum, as well as a decrease in the levels of 5-HT and BDNF in the brain. The NAO treatment improved the intestinal flora and increased the short-chain fatty acid (SCFA) content in the cecum, in addition to restoring the levels of the molecular markers of depression mentioned above. Additionally, the effects of the gut microbiota in conveying NAO antidepressant properties were explored by FMT.

## 2. Results

### 2.1. Effects of NAOs on Body Weight in CRS-Induced Mice

**Figure 1:**
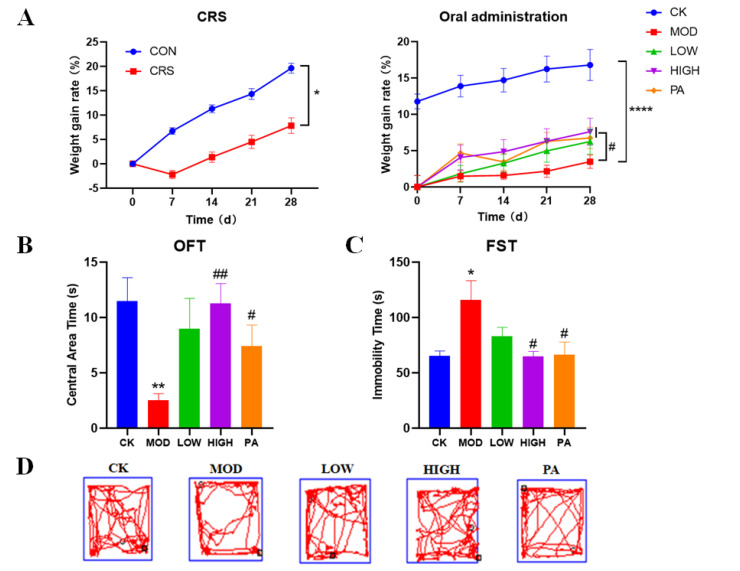
Effects of NAOs on body weight, OFT, and FST in CRS-induced mice. (**A**) The CRS mice were repeatedly placed in a plastic restrainer customized with a 50 mL centrifuge tube for 4 h (from 10:00 to 14:00) every day for 28 consecutive days. During the restraint stress period, the CRS mice were deprived of food and water. Mice in the control group had free access to water and food without any restraint. After establishing the depression model on day 28, The mice in the low-concentration NAO group (LOW) and HIGH groups were supplied daily with NAOs at doses of 100 and 200 mg/kg based on their body weight, respectively. The mice in the PA group were supplied with paroxetine at a dose of 10 mg/kg. The mice in the CK and CRS groups received sterilized double-distilled water through oral gavage in the same volume as other groups. *n* = 10–12 per test. (**B**) Central area time over 4 min in the OFT. *n* = 4–5 per test. (**C**) Immobility time in FST. *n* = 4–5 per test. (**D**) Representative locomotion traces in OFT. Two-way ANOVA tests were used to analyze the weight gain rate, and unpaired *t* tests were used to analyze the other data. Data are presented as mean ± SEM. * *p* < 0.05, ** *p* < 0.01, **** *p* < 0.0001 vs. control, # *p* < 0.05, ## *p* < 0.01 vs. model group.

CRS induction led to slow weight gain in the mice. During the experiment, the mice were weighed every 7 days. After 28 days of CRS induction, the body weight of the CRS-induced group and the control group changed significantly. CRS induction could significantly decelerate the weight gain of the mice. Based on the experimenter’s observation, the mice in the CRS-induced group showed huddling and less movement, were timid and easily frightened and irritated, and had rougher hair and a loss of interest. All of these results indicated that the modeling was successful. After 28 days of administration, the slow weight gain caused by the CRS induction was significantly reversed in the high-concentration NAO group (HIGH) group and paroxetine (PA) groups (Figure 1A).

### 2.2. Effects of NAOs on CRS-Induced Mice in the Open Field Test (OFT)

The OFT was conducted to assess depression-like behavior. In the OFT, the model (MOD) group spent less time in the center of the OFT area, while the HIGH and PA groups spent more time there (Figure 1B).

### 2.3. Effects of NAOs on CRS-Induced Mice in the Forced Swimming Test (FST)

Next, the FST was used to measure behavioral despair/helplessness. In the FST, the mice in the MOD group exhibited a significantly longer immobility time compared with the control (CK) group. However, this was reversed by the gavage of high-concentration NAO and paroxetine, and the immobility time returned to the level of controls (Figure 1C).

### 2.4. Effect on the Weight Gain Rate after Terminating the Gavage of NAOs

**Figure 2:**
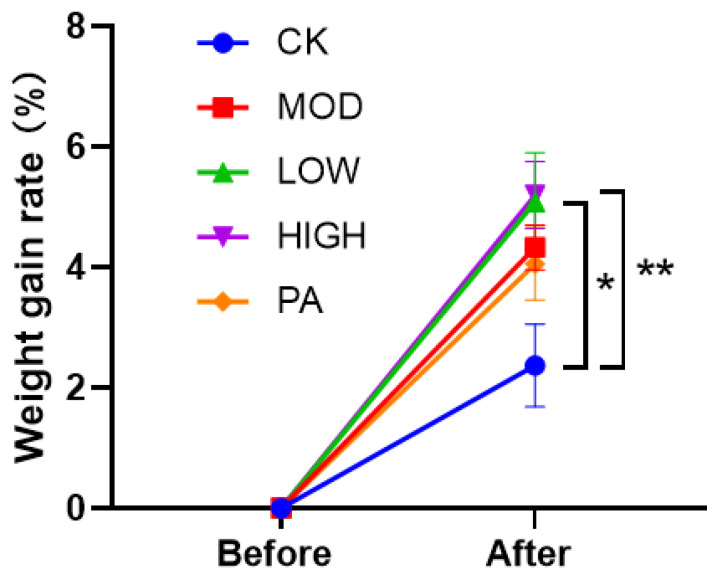
Weight gain rate 7 days after terminating the restraints and administrations. Data are presented as mean ± SEM. * *p* < 0.05, ** *p* < 0.01 vs. control group.

Seven days after terminating the restraints and administrations, the weight growth rate of the mice fed with NAOs was higher than that of the MOD group. The weight growth rate of the mice fed with NAOs was higher than that of the PA group. However, the weight growth rate of the mice fed with paroxetine (PA group) was lower than that of the MOD group (Figure 2).

### 2.5. Oral Administration of NAOs Modulated the Gut Microbiota Composition of Depressed Mice

**Figure 3:**
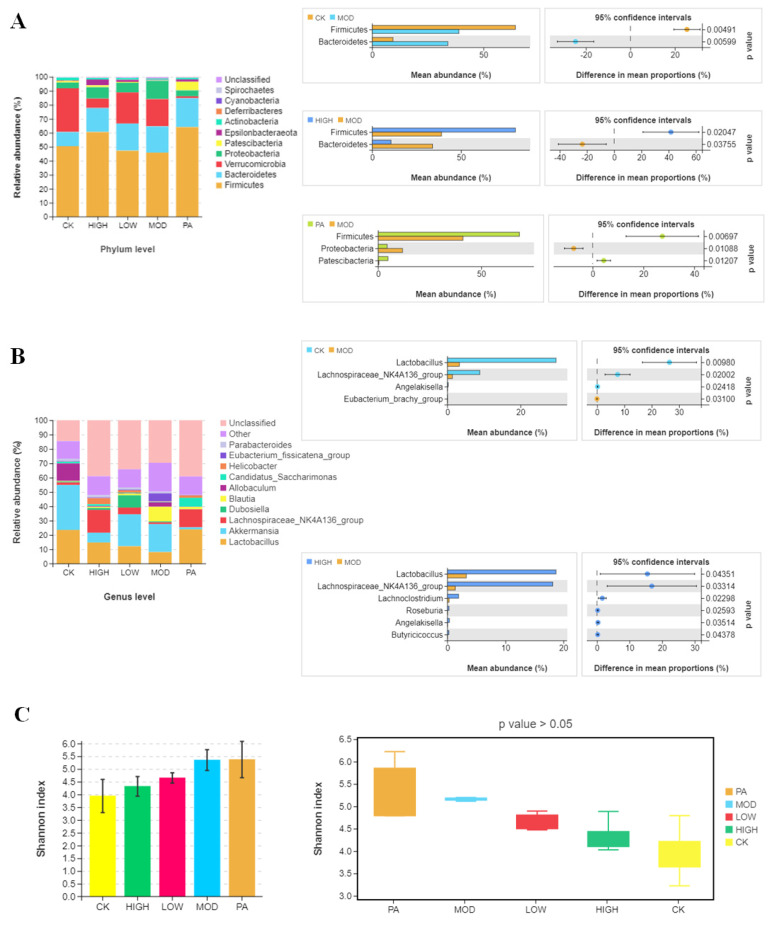

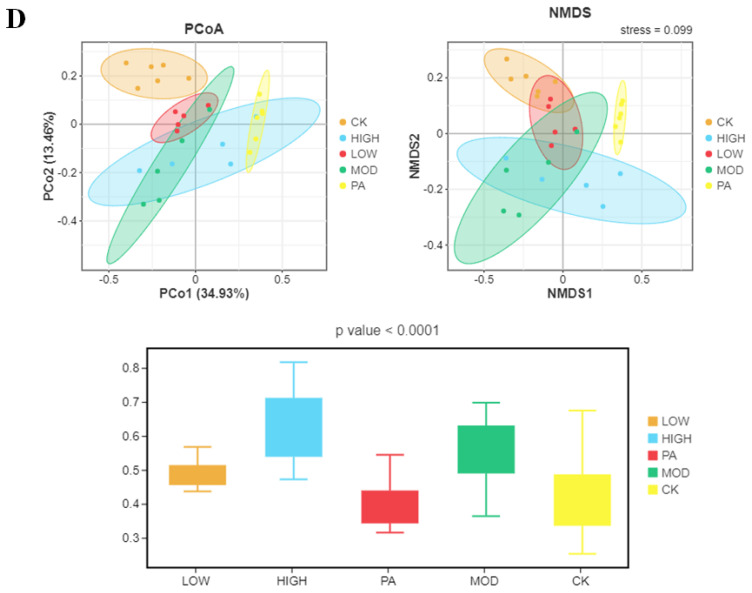
Gut microbiota diversity. (**A**) Relative abundance of the cecal microbiota on the phylum level and mean abundance of marker species in each group. The *p* values are based on Welch’s *t* test. (**B**) Relative abundance of the cecal microbiota on the genus level and mean abundance of marker species in each group. The *p* values are based on Welch’s *t* test. (**C**) The α-diversity analysis and Shannon diversity index. The *p* values are based on Kruskal–Wallis. (**D**) Effects of NAOs on β-diversity of intestinal microbes. PCoA and NMDS score plots based on Bray (OTU) (*p* < 0.0001).

Considering that changes in body weight were linked with the host’s gut microorganisms, we assessed the effects of NAOs on the gut microbiota composition of the depressed mice. We analyzed the relative abundance of the dominant taxa identified by sequencing in all the groups. The microbial composition of cecal contents in different groups exhibited significant differences on multiple taxonomical levels. Firstly, the microbial community structure of each group was presented on the phylum level. The primary phyla were Firmicutes, Bacteroidetes, Verrucomicrobia, and Proteobacteria, accounting for >90% of the microbial compositions in all the samples. As shown in Figure 3A, the relative abundances of Firmicutes and Bacteroidetes were significantly different between the CK group and the MOD group. Secondly, compared with the MOD group, the NAO administration evidently ameliorated the dysbiosis of Bacteroidetes induced by CRS and increased the relative abundance of Firmicutes. Moreover, compared with the MOD group, paroxetine administration increased the relative abundance of Firmicutes and decreased the relative abundance of Proteobacteria. To further identify the cecal microbial community alterations, the microbial community structure of each sample was then presented on the genus level. The gut microbiota of the top 10 genera with a higher relative abundance are shown in Figure 3B. Compared with the CK group, the relative abundances of *Lactobacillus* were decreased in the MOD group, whereas the oral administration of NAOs could attenuate the changes in these microbes.

Moreover, the α-diversity analysis demonstrated no significant differences between the groups (Figure 3C). In addition, principal coordinate analysis (PCoA) and nonmetric multidimensional scaling (NMDS) were used to discover the overall structural changes in the gut microbiota among the five groups. The results exhibited an apparent clustering in the microbial composition of each group (Figure 3D). In particular, the PA group could be clustered in the smallest area alone. The difference between the LOW and CK groups was smaller than that between the CRS-induced mice and normal ones, which indicated that the NAOs ameliorated the microbial composition of the CRS-induced mice.

### 2.6. Oral Administration of NAOs Increased the Levels of Short-Chain Fatty Acids (SCFAs) in the Feces of Depressed Mice

**Figure 4:**
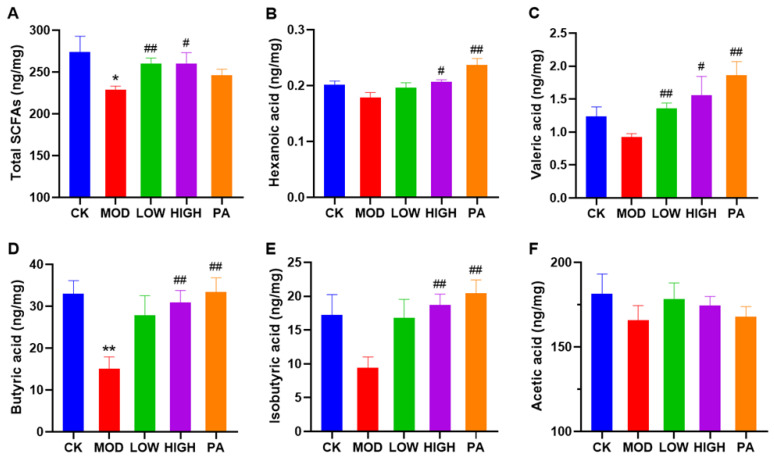
Effects of NAOs on the fecal SCFAs in CRS mice. (**A**) Total SCFAs, (**B**) hexanoic acid, (**C**) valeric acid, (**D**) butyric acid, (**E**) isobutyric acid, and (**F**) acetic acid, *n* = 4–5 per test. Unpaired *t* tests were used to analyze the data. Data are presented as mean ± SEM. * *p* < 0.05, ** *p* < 0.01 vs. control group; # *p* < 0.05, ## *p* < 0.01 vs. model group.

The SCFA levels in the cecum contents were obtained by GC-MS analysis (Figure 4). The level of total SCFAs, as well as hexanoic acid, valeric acid, butyric acid, isobutyric acid, and acetic acid, in the cecum contents of the MOD group were reduced compared with the CK group. However, the oral administration of NAOs reversed the reduction in the total SCFAs. We also found that the levels of total SCFAs, hexanoic acid, valeric acid, butyric acid, and isobutyric acid were increased in the cecum contents of the PA group compared with the MOD group. Overall, the oral administration of NAOs could effectively reverse the decrease in SCFAs in the cecum contents of the CRS-induced depressed mice.

### 2.7. Effect of NAOs on the Expression of IL-18 and 5-HT in the Serum of Mice

**Figure 5:**
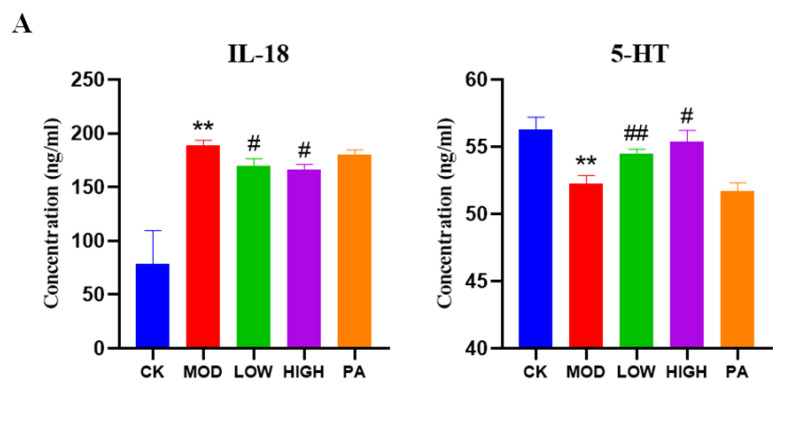

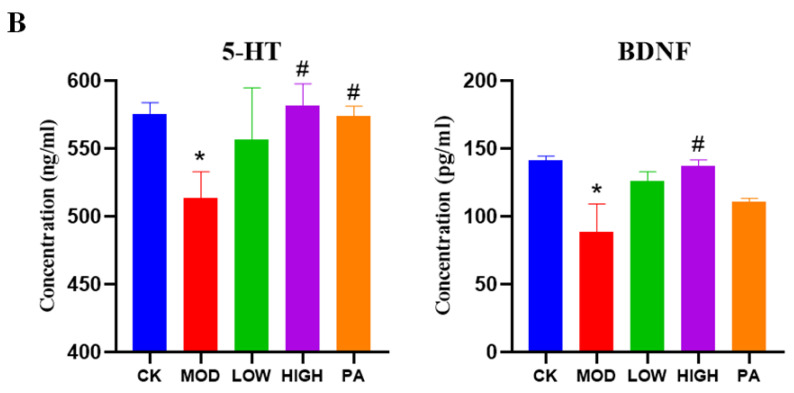
Biochemical assays in the serum and whole brain. (**A**) IL-18 and 5-HT levels were detected by ELISA in the serum. *n* = 4–5 per test. (**B**) 5-HT and BDNF in the brain were detected by ELISA. *n* = 4–5 per test. Unpaired *t* tests were used to analyze the data. Data are presented as mean ± SEM. * *p* < 0.05, ** *p* < 0.0 vs. control, # *p* < 0.05, ## *p* < 0.01 vs. model group.

Given the anti-inflammatory effect of NAOs, we investigated their effects on IL-18 and 5-HT in the serum of the mice. As shown in Figure 5A, the IL-18 levels were significantly increased, whereas the 5-HT levels were notably decreased in the CRS group compared to the control group. We found that the levels of IL-18 were reduced significantly in the serum compared with the MOD group, while the levels of 5-HT were significantly recovered.

### 2.8. Effect of NAOs on the Expression of 5-HT and BDNF in the Whole Brain of Mice

To further explore the effects of NAOs, the expression of 5-HT and BDNF in the whole brain was determined (Figure 5B). The expression of 5-HT and BDNF was decreased in the MOD group relative to the CK group. However, the levels of 5-HT and BDNF were significantly increased in the HIGH group compared with those in the MOD group.

### 2.9. FMT Confirmed the Involvement of Gut Bacteria in the NAO Modulation of Weight Changes, as Well as Anxiety- and Depressive-Like Behavior

**Figure 6:**
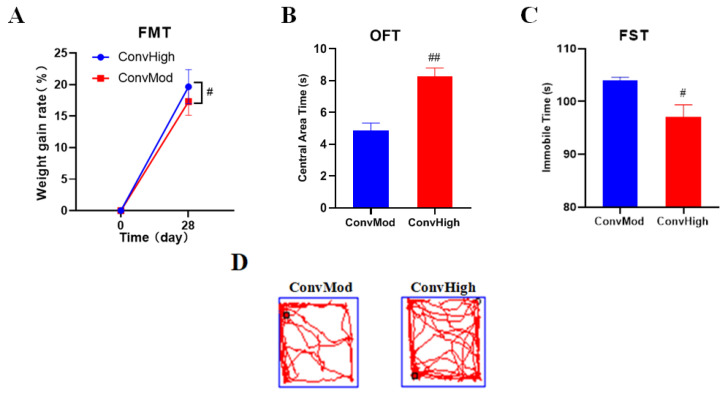
Effects of NAOs on body weight, OFT, and FST in FMT mice. (**A**) Weight measurements of FMT mice. *n* = 4–5 per test. (**B**) OFT of FMT mice. *n* = 4 per test. (**C**) FST of FMT mice. *n* = 4 per test. (**D**) Representative locomotion traces in OFT. # *p* < 0.05, ## *p* < 0.01 vs. model group.

To further verify the antidepressant effect of NAOs caused by gut microbiota modulation, we performed FMT. During the experiment, the mice were weighed every 7 days. After 28 days of gavage, the weight of the ConvHigh group was significantly higher than that of the ConvMod group (Figure 6A). To investigate the impact of FMT on the mice’s anxiety and depression behavior, we chose OFT and FST. As shown in Figure 6B, the ConvMod group spent less time in the center of the OFT area, indicating their higher anxiety, compared to ConvHigh group, and the immobile time of the ConvMod group was remarkably higher than that of the ConvHigh mice, indicating their higher depression.

## 3. Discussion

Various non-digestible and less toxic oligosaccharides, including inulin, xylooligosaccharides, fructooligosaccharides (FOS), galactooligosaccharides (GOS), and isomaltoses, have entered the market in recent years [35]. NAOs are considered as potential interesting candidates due to their prebiotic benefits. Recent studies showed that the prebiotic effects of NAOs were better than those of other non-digestible oligosaccharides, such as FOS and GOS. NAOs extracted from seaweed are different from terrestrial plant oligosaccharides and, thus, have new prebiotic activities. A previous study demonstrated that neoagarotetraose remarkably reduced the accumulation of harmful metabolic byproducts and protected the epithelial integrity of the gut [36]. For example, compared with FOS, NAOs showed better prebiotic effects and earlier immune reactions, benefiting the colonic microflora balance [31]. Additionally, studies have shown that NAOs can significantly extend the observed and maximum lifespans of male *Drosophila melanogaster* and improved their intestinal immunity [37]. Therefore, NAOs have great potential to be applied as nutraceutical and functional food ingredients. However, our study showed, for the first time, that NAOs can be used for the treatment of depression, since they improved anxiety and depressive-like behavior in the CRS-induced model of depression.

The change in the CRS mice was directly reflected in their body weight. The gavage of high-concentration NAOs reversed the weight loss of the CRS mice. Meanwhile, the success of the NAOs in curing the CRS mice was proven by behavioral tests. According to the usual findings of antidepressant experiments, changes in body weight are generally regulated by gut microorganisms. Therefore, we hypothesize that the antidepressant effects of NAOs were mediated through the modulation of the gut microbiota.

Accumulating evidence highlights the potential association between the gut microbiota and depression via the microbe–gut–brain axis [38,39]. Research has shown that disruptions in the intestinal microbiota in later life, triggering an inflammatory cascade through the gut–brain axis, can induce significant inflammatory effects on the CNS, which influence cognitive function and the expression of anxiety and depression (mood). The gut microbiota plays an important role in host metabolism, and gut microbiota dysbiosis is often associated with the dysregulation of the microbiota–host co-metabolism [40,41]. In addition, more evidence has revealed that metabolic signature perturbations associated with changes in the gut microbiota composition are important risk factors for the development of diseases [42]. Our study demonstrated that when the gut microbiota was altered, the fecal metabolic phenotype in the depressive condition was also altered. To explore the association between the antidepressant effect of NAOs and the regulation of the gut flora, we examined the gut microbiota composition and structure via 16S rRNA gene sequencing. The α-diversity analysis demonstrated no significant differences in species richness and fecal microflora diversity between the groups, which is consistent with the results of Naseribafrouei [43] and Song [44]. However, in one study, chronic social frustration stress significantly improved the reduced α-diversity of the gut microbiota in antibiotic-treated mice [45]. Fecal microbial diversity (estimated using the Shannon index) has been reported to be unexpectedly higher in patients with major depressive disorder (MDD). Although greater gut microbial diversity is potentially beneficial for human health, its role in CNS function is still controversial and must be further verified [46]. In the β-diversity analysis, the results revealed a clear difference between the model and other groups, showing obvious changes in the microbial community structure after chronic stress. The results of the LOW, HIGH, and PA groups significantly deviated from those of the MOD group, showing that paroxetine and NAOs regulated the fecal microbial structure of the CRS-induced mice. The statistical analyses employing the Bray and unweighted UniFrac distance indices showed significant differences between the MOD and CK groups, the MOD and NAO groups, and the MOD and PA groups. The results showed that depression significantly altered the host’s intestinal flora. However, the NAOs could alleviate depression by increasing the amount of beneficial bacteria and reducing that of harmful bacteria.

Subsequently, a more detailed analysis of the microbial community composition was conducted. In this study, the gut flora of the mice changed under stress and was characterized by the increased abundance of pathogenic bacteria and decreased abundance of beneficial bacteria. Specifically, the abundance of Bacteroidetes increased in the model group, whereas the abundance of Firmicutes decreased. This result has also been observed in the gut flora of patients with severe depression [46]. Treatment with paroxetine and NAOs reversed this phenomenon. Furthermore, we found that the relative abundances of *Ruminococcus*, *Blautia*, *Eubacterium*, and *Parasutterella* were significantly increased in the cecal contents of the MOD group mice with respect to those in the CK group, whereas the relative abundances of *Lactobacillus* and Lachnospiraceae were significantly reduced. However, the abundances of these genera were reversed by the NAO and paroxetine administration. Consistent with our findings, an increased level of *Ruminococcus* was also reported in subjects with psychiatric disease [47]. *Lactobacillus* belongs to the lactic acid bacteria and colonizes several parts of the body, including the skin, vagina, and entire gastrointestinal tract, starting with the oral cavity [48]. Patients with MDD tended to have lower *Lactobacillus* counts in their fecal samples compared with those of the controls [49]. Within the genus *Lactobacillus*, there are broad spectra of species with diverse physiological properties. Most *Lactobacillus* strains have some beneficial applications. Previous studies have suggested that the administration of *Lactobacillus* has an antidepressant effect on stress-induced depressive disorder [50,51]. In the current study, we also found that NAO administration significantly inhibited the decrease in the relative abundance of *Lactobacillus* in depressed mice, which was likely beneficial for the improvement of their depression.

Zhang et al. [52] studied the fermentation behavior of NAOs derived from *Gracilaria lemaneiformis* and found that NAOs can increase the concentrations of SCFAs and modulate the composition and diversity of gut microorganisms by increasing the abundance of Firmicutes and Actinobacteria and reducing that of potential pathogenic bacteria. Our study conclusions also agree with some of these studies. There was a significant increase in the levels of SCFAs in the NAO group compared with the MOD group, whereas paroxetine could not increase the level of SCFAs remarkably. These findings may be related to the changes in the gut microbiota. Bendtsen [53] also reported that the expression of various species of Lachnospiraceae and Ruminococcaceae in mice was correlated with behavioral changes induced by stress. The Lachnospiraceae family, which includes *Roseburia*, *Blautia*, and *Lachnospiraceae incertae sedis*, participates in the breakdown of carbohydrates into SCFAs [54]. A decrease in these fermentation-related bacteria precipitates a decline in SCFA production, which in turn causes intestinal barrier dysfunction [55]. Evidence also suggests that brain function and behavior are influenced by SCFAs. SCFAs are involved in gastrointestinal physiology, immune function, host metabolism, and the development and homeostasis of the CNS [56]. SCFAs can stimulate enterochromaffin cells to produce 5-HT [57] and, thus, affect the mood of the brain. In addition, SCFAs can interact with nerve cells by stimulating the sympathetic and autonomic nervous system via G-protein-coupled receptor 41 (GPR41) and GPR43 [58]. Furthermore, SCFAs can promote the growth and development of brain microglia, which are the most important immune barrier of the CNS, maintain cell homeostasis, and enhance brain immune defense function [59].

In addition, a decrease in the diversity of the intestinal microflora, reduction in probiotics, and intestinal microflora dysregulation lead to a decrease in 5-HT [60]. Abnormalities in central 5-HT neurotransmission are considered to play a key role in the pathogenesis of depression [61]. Meanwhile, 5-HT is the major target of newer antidepressant drugs, such as selective serotonin re-uptake inhibitors. NAOs increase the levels of 5-HT in the brain, enabling their use as antidepressants. In addition, numerous pieces of evidence suggested that BDNF is one of the key factors in the pathogenesis of depressive disorders, as the BDNF concentrations decreased with increasing depression severity [62]. In animal studies, the infusion of BDNF into the midbrain area and hippocampus produced antidepressant-like behavioral effects in rats. Human studies reported that a decreased protein expression of BDNF was discovered in the brains of depressed suicide victims [63]. However, increased BDNF levels can support the mechanism through which antidepressants confer their beneficial functions [64]. These findings were consistent with our results, which indicated that 5-HT and BDNF were related to depression. Furthermore, increasing the levels of 5-HT and BDNF could alleviate depression, the mechanism of which might be related to the gut microbes [65]. Meanwhile, chronic inflammation has been suggested to be an important mechanism related to depression. IL-18 is a newly discovered proinflammatory cytokine, and higher circulating IL-18 has also been reported in patients with depression [10].

It is important to further validate the authentic links between the gut microbiota and its effects on the host metabolic phenotype in GF mice. There is a notion that FMT may also be effective in alleviating some psychiatric disorders. Previous results suggested that FMT reduced depressive symptoms in patients with irritable bowel syndrome (IBS) after 4 weeks of treatment [66]. Studies on GF mice have indicated that gut microbiota transplantation can affect not only their behavior but also their hypothalamic–pituitary–adrenal stress response and BDNF levels in the hippocampus [67]. Our experiment confirmed the involvement of the gut microbiota in the NAO modulation of weight changes, as well as anxiety- and depressive-like behavior. Considering the potential side effects of paroxetine, including lethargy and dystonia, Hong [30] first reported the safety of NAOs. The results showed that after 5000 mg/kg BW/day of NAO intake, SD rats and beagles showed no symptoms of toxicity, indicating the safety and potential applications of NAOs. This probably explains why the weight gain rate of the mice fed with paroxetine was lower than that of the MOD group after 7 days of drug withdrawal, whereas the weight grain rate of the mice fed with NAOs was higher than that of the MOD group (Figure 2). Due to the termination of restraints, the body weight, which was previously suppressed, was increased and rebounded. Thus, it is understandable that the weight gain rate of the mice in the CRS group was higher than that of the CK group. However, only the weight gain rate of the HIGH group was significantly higher than that of the CK group. While paroxetine relieved depression, the side effects also caused a certain degree of damage to the body [11]. Therefore, after stopping the drug, the body recovered more slowly, which was reflected in the change in body weight. Our experiments showed that mice can tolerate up to 37 g/kg of NAOs (data not shown). The safety of NAOs indicates their promising potential as antidepressants or functional foods to act against depression.

Taken together, we demonstrated that NAOs’ antidepressant effect may be exerted through the increase in beneficial bacteria and decrease in harmful bacteria, thus achieving an antidepressant effect. The increase in beneficial bacteria can increase the SCFA content and 5-HT and BDNF levels in the brain and decrease the IL-18 levels through the brain–gut axis. Simultaneously, these factors exerted antidepressant effects.

## 4. Materials and Methods

### 4.1. Preparation and Determination of NAOs

The NAOs were prepared by enzymatic hydrolysis from algae polysaccharides using recombinant agarase Aga3027 derived from a marine bacterium, *Flammeovirga* sp. OC4, in our previous study. The neoagaro-oligosaccharides, the products hydrolyzed by agarases, were identified as neoagarotetraose and neoagarohexaose by thin-layer chromatography and ion chromatography [68]. Overall, the NAOs contained 95% active ingredients, 3.5% moisture, and 1.5% ash. Among the active ingredients, neoagarotetraose accounted for 55% and neoagarohexaose accounted for 45%. The NAOs were dissolved in sterile double-distilled water and filtered through a 0.22 μm filter membrane before use.

### 4.2. Animals

Five-week-old male C57BL/6J mice were chosen [69] and purchased from Shanghai SLAC Laboratory Animal Co., Ltd. (Shanghai, China). The mice were habituated in a temperature-controlled animal facility with a 12/12 h light/dark cycle for 7 days and had free access to water and food. They were stroked by the experimenter for 3 min/day to reduce the operational impact. Efforts were made to minimize animal suffering as much as possible.

Six-week-old male C57BL/6JGpt (GF) mice, purchased from Gem Pharmatech. Co., Ltd. (Jiangsu, China), were selected as the recipient mice for the FMT experiments.

The experimental program was approved by the Institutional Committee on the Care and Use of Animals of the Third Institute of Oceanography, Ministry of Natural Resources (TIO-IACUC-05-2021-11-08). All the animals received humane care according to the National Institutes of Health (Bethesda, MD, USA) guidelines.

### 4.3. CRS Procedure

After their habituation, all the mice were divided into two groups randomly (a control group and CRS-induced group). The mice in the CRS-induced group were repeatedly placed in a plastic restrainer customized with a 50 mL centrifuge tube for 4 h (from 10:00 to 14:00) every day for 28 consecutive days. During the restraint stress period, the mice in the CRS-induced group were deprived of food and water. The mice in the control group had free access to water and food without any restraint [70].

### 4.4. Experimental Groups and Drug Administration

After establishing the depression model, the mice in the control group served as the CK group. Meanwhile, the mice in the CRS-induced group were randomly divided into four equal groups: a model group, low-concentration NAO group, high-concentration NAO group, and paroxetine group.

The mice in the LOW and HIGH groups were supplied daily with NAOs at doses of 100 and 200 mg/kg based on their body weight, respectively. The mice in the PA group were supplied with paroxetine at a dose of 10 mg/kg. The mice in the CK and CRS groups received sterilized double-distilled water through oral gavage in the same volume as other groups.

**Figure 7:**
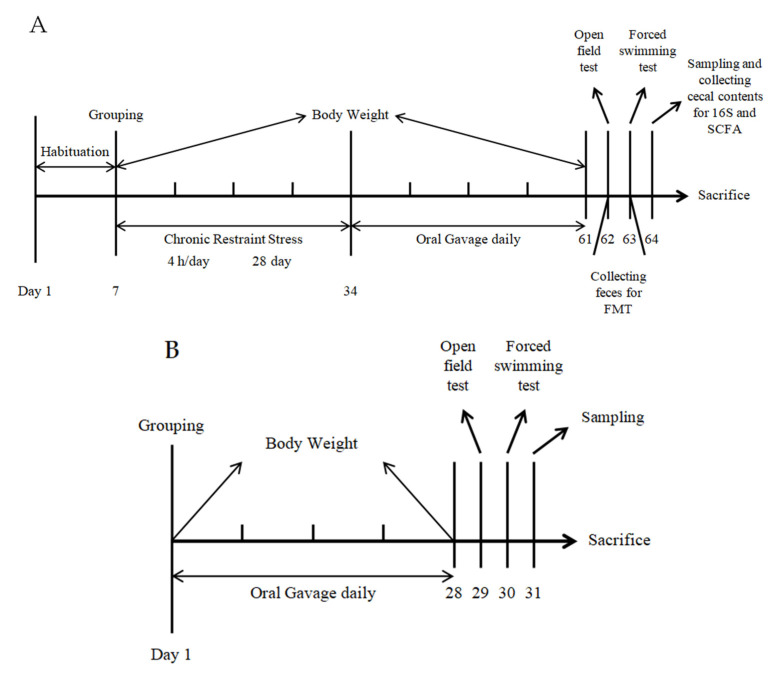
Experimental procedure. (**A**) Schematic of the total experimental procedures. (**B**) Schematic of the FMT experiment.

After 28 days of oral gavage, the mice in all the groups were randomly divided into two equal groups. Some of the mice were subjected to behavioral experiments, whereas the remaining mice did not receive gavage for a week. During the withdrawal period, the mice were free to eat and drink. Seven days later, their body weight was measured, and behavioral experiments were performed (Figure 7A).

### 4.5. OFT

The OFT is a classic behavioral test used to evaluate the level of anxiety in animals [71]. The open field apparatus consisted of a 100 cm × 100 cm gray box with 40 cm walls. Each animal was gently placed in the central square to acclimate for 1 min and observed for 4 min using a joint open field experiment video analysis system (ZH-OFT, Anhui Zhenghua Biological Instrument Equipment Co., Ltd., Anhui, China). The apparatus was cleaned with 75% ethanol after each trial.

### 4.6. FST

The FST is a well-recognized paradigm for screening the antidepressant-like effects of drugs [72]. The apparatus consists of a cylinder (29 cm high and 11 cm in diameter). Before the test, the cylinder was filled with fresh water at a temperature of 25 ± 1 °C [73]. Then, the animals were gently immersed in it. The immobility time of each animal was recorded using an autovision camera, and the last 4 min period was analyzed using an FST video analysis system (ZH-FST, Anhui Zhenghua Biological Instrument Equipment Co., Ltd., Anhui, China).

### 4.7. Gut Microbiota Analysis

Cecal contents were collected after sacrificing the animals to perform 16S rRNA gene sequencing, and fecal contents were collected before sacrificing the animals to determine the SCFAs. Genomic DNA was isolated from the cecal contents of the mice. The conserved regions of the 16S rRNA gene were amplified using specific primers. Then, the polymerase chain reaction amplification products were recovered from the gel and quantified with a QuantiFluor™ fluorometer (Promega, Madison, WI, USA). The purified amplification products were mixed in equal amounts, and the sequencing adapters were connected to construct a sequencing library, which was sequenced on an Illumina PE250 (San Diego, CA, USA). Briefly, the sequences with a ≥97% similarity were sequenced on the Illumina platform according to the same operational taxonomic units. Alpha diversity was applied when analyzing the complexity of the species diversity for a sample using seven indicators, including Sob, Chao1, ACE, Shannon, Simpson, PD-tree, and Pielou. Generally speaking, the calculation of the community beta diversity includes two parts: the species change (how much) and species generation (whether or not). Based on these two important indicators, we used weighted the Unifrac, unweighted Unifrac, Jaccard, and Bray to conduct the subsequent beta diversity analysis.

For the LC-MS/MS analysis, a sample of another part of the cecal contents was treated as follows: Add 300 μL of 50% acetonitrile, vortex for 1 min, sonicate for 30 min at 4 °C, and centrifuge at 12,000 rpm for 15 min at 4 °C. Then, add 3-NPH (200 mM) and EDC (120 mM, containing 6% pyridine) solution (2:1:1 *v*/*v*/*v*), vortex for 1 min (2:1:1 *v*/*v*/*v*), react at 40 °C for 1 h, and oscillate once every 5 min. After the reaction was completed, the supernatant was collected by centrifugation at 12,000 rpm for 15 min at 4 °C and diluted 100 times with 50% acetonitrile water using the machine for the LC-MS/MS analysis.

### 4.8. Biochemical Assays

All the mice were sacrificed at the end of the experiment. The serum and tissues, including brain and cecal contents, were collected and stored at −80 °C as quickly as possible. The supernatant of the serum was collected by centrifugation at 3000 rpm for 20 min at 4 °C. The levels of 5-HT and IL-18 were determined using an ELISA Kit (Shanghai YuanJu Biotechnology Center, Shanghai, China). Then, they were stored at −80 °C for subsequent analysis.

Brain homogenate was prepared by mixing cold 0.9% saline (XiLong Scientific Co., Ltd., Guangdong, China) and brain tissue. The ratio of brain to saline was 1:4, as recommended by Fernandes [74], to avoid changes in the solution concentration. The temperature of the solution surrounding the samples changed by <1 °C during the experiments. The sonication treatment time was 35 s (pulse duration of 3 s on-time and 5 s off-time). The supernatant of the brain homogenate was collected by centrifugation at 8000 rpm for 10 min at 4 °C. The levels of 5-HT and BDNF were determined using an ELISA Kit (Shanghai YuanJu Biotechnology Center). Then, they were stored at −80 °C for subsequent analysis.

### 4.9. FMT

All the recipient mice were divided into two groups (ConvMod and ConvHigh). The mice in the MOD and HIGH groups provided fecal microbiota as the donor mice. Several fecal pellets from these two groups of mice were collected using sterile tubes and blended with 4 mL of sterile 30% glycerin. Then, the sample was stirred, and the filtrate was collected after being filtered twice through a 70 μm filter. Afterward, the sample was stored at −80 °C. For each donor mouse, 200 μL of bacterial suspension was transplanted into each of the recipient mice by gavage every day for 28 consecutive days (Figure 7B) [65].

### 4.10. Statistical Analysis

All the data, presented as mean ± SEM, were calculated and demonstrated using GraphPad Prism 8 (San Diego, CA, USA). One-way analysis of variance (ANOVA) and Dunnett’s post hoc test for multiple comparisons were applied to compare the behavioral test results and to establish statistically significant differences. For the gut microbial data analysis, the Kruskal–Wallis test with Bonferroni’s correction was performed to determine significant changes in the microbiota species. The differences in the α-diversity index between groups were evaluated using the Wilcoxon test, and the results of the β-diversity index were analyzed by the nonparametric test analysis of similarities.

## 5. Conclusions

Our study investigated the CRS-induced depression model and the associated changes in the gut microflora of mice treated with NAOs and confirmed that the antidepressant effect of NAOs is linked to the remodeling of the gut flora. The results showed that the NAOs mainly improved the level of beneficial bacteria and reduced the level of harmful or inflammatory bacteria. Compared with paroxetine, NAOs may have a better safety profile and fewer side effects. These findings indicate the promising potential of NAOs as antidepressants or functional foods for depression.

## Data Availability

Not applicable.

## Abbreviations

CK group	Control group
NAOs	Neoagaro-oligosaccharides
OFT	Open field test
FST	Forced swimming test
SCFAs	Short-chain fatty acids
FMT	Fecal microbiota transplantation
BDNF	Brain-derived neurotrophic factor
MOD group	Model group
LOW group	The low-concentration NAOs group
HIGH group	The high-concentration NAOs group
PA group	Paroxetine group
ConvHigh	Microbiota depleted mice that were conventionalized with cecal samples from HIGH group mice
ConvMod	Microbiota depleted mice that were conventionalized with cecal samples from MOD group mice
